# Knowledge, attitude, and practice of self-medication among adults in the central region of Saudi Arabia: a cross-sectional study

**DOI:** 10.3389/fpubh.2026.1802800

**Published:** 2026-03-31

**Authors:** Rawand J. Aldali, Emadeldin M. Elsokkary, Huda B. Almousa, Yousef H. Alzahrani, Haneen E. Elsokkary, Jehad A. Aldali

**Affiliations:** 1 School of Chemistry, University of Bristol, Bristol, United Kingdom; 2 Department of Psychology, College of Social Sciences, Imam Mohammad Ibn Saud Islamic University (IMSIU), Riyadh, Saudi Arabia; 3 College of Medicine, King Saud University, Riyadh, Saudi Arabia; 4 Faculty of Medicine, Horus University in Egypt (HUE), New Damietta, Egypt; 5 Department of Pathology, College of Medicine, Imam Mohammad Ibn Saud Islamic University (IMSIU), Riyadh, Saudi Arabia

**Keywords:** adult population, drug utilization, health-seeking behavior, personal experience, Saudi Arabia, self-medication

## Abstract

**Background:**

Public health concerns about self-medication (SM) are growing worldwide. SM can manage minor ailments, reduce healthcare system burdens, and empower patients to manage their health, but it is crucial to understand the potential risks and consequences associated with improper use. Its misuse is also dangerous. This study seeks to assess the knowledge, attitudes, and practices concerning SM among the general population in the central region of Saudi Arabia.

**Methods:**

A cross-sectional study utilized questionnaires to gather data. The distribution started on 6 June 2025 and ended on 11 December 2025.

**Results:**

The questionnaire was completed by 384 people. Females had slightly higher knowledge Median = 2.50, IQR = 2.17–2.67 and 40–50-year-olds had the highest knowledge. The most common reason for SM was headache (336; 87.5%), followed by cold and flu (315; 82.0%) and fever (283; 73.7%). Stomach-ache (221; 57.6%), insomnia (184; 47.9%), and menstrual symptoms were moderately common. The most common was minor illness (329; 85.7%), indicating that 9SM is acceptable for simple, self-limiting health issues. Over half of participants cited lack of time to see a doctor (210; 54.7%) and pharmacological knowledge (208; 54.2%) as motivators. Personal experience (324; 84.4%) was the most influential factor, indicating that people SM based on their knowledge of previous illnesses and treatments. Family members’ opinions had a significant impact (272; 70.8%), reflecting family dynamics and social support in the cultural context.

**Conclusion:**

The findings indicate that SM is a common health behavior, largely associated with perceptions of minor illness, convenience, prior personal experience, and social influences. No statistically significant differences were observed across several demographic comparisons in knowledge and attitude scores. These findings should be interpreted cautiously, particularly given the absence of multivariable analysis and the overrepresentation of younger adults and university graduates in the sample.

## 1 Introduction

Self-medication (SM) is a growing global public health concern. SM can be beneficial in some contexts, such as managing minor ailments, reducing the burden on healthcare systems, and empowering patients to take charge of their own health (1). It also carries significant risks when misused. Inappropriate SM may result in serious health risks, including incorrect self-diagnosis, masking of severe conditions, drug misuse, inappropriate drug selection or dosage, harmful drug interactions, delays in seeking appropriate medical care, and the potential for dependency and abuse (2–5).

In recent years, SM has become increasingly common worldwide, driven by a range of individual, social, and systemic factors. Studies have highlighted influences such as a person’s education and age, family beliefs, existing legislation on drug sales, pharmaceutical advertising, prior experiences with illnesses, the perceived severity of symptoms, availability of leftover prescription medications at home, and economic circumstances. In addition, broader factors like easy access to over-the-counter (OTC) drugs, rising healthcare costs, long waiting times in clinics and hospitals, and widespread access to health information online have further contributed to the growth of SM practices (6, 7). These factors are especially important in low- and middle-income countries, where healthcare systems may be overworked and rules are not always followed (8). In such settings, community pharmacies frequently serve as the first point of care, allowing easy access to a wide range of medications without prescriptions.

Among the most pressing concerns related to SM is the misuse of antibiotics. The improper and widespread use of antibiotics without professional guidance has been directly linked to the emergence and spread of antibiotic-resistant bacteria, a phenomenon that poses a serious threat to global health (9, 10). The World Health Organisation (WHO) has recognized antimicrobial resistance as one of the top ten global health threats, warning that the continued misuse of antibiotics could lead to a post-antibiotic era in which common infections become deadly once again (11).

Within the Middle East, and particularly in countries such as Saudi Arabia, SM is a common practice. Cultural factors, high levels of trust in pharmacists, and lenient regulations around medication sales contribute to the widespread use of non-prescription medications (12). Studies have shown that many individuals in Saudi Arabia SM, based on past experiences or the advice of family and friends, often do so without fully understanding the potential risks (13). While research has explored SM behaviors in various regions of the country, there remains a lack of comprehensive data specific to the central region, where differing levels of urbanization, healthcare accessibility, and education may significantly influence health behaviors.

Understanding the knowledge, attitudes, and practices (KAP) of the public regarding SM is essential for forming effective health policies and public awareness campaigns. Assessing these factors can help identify gaps in health literacy, areas of public misunderstanding, and patterns of risky behavior, all of which are crucial for designing interventions that promote the rational and safe use of medications.

Therefore, this study aims to evaluate the knowledge, attitudes, and practices related to SM among the general population in the central region of Saudi Arabia.

## 2 Methods

### 2.1 Study design and settings

A cross-sectional, descriptive study was conducted to assess the knowledge, attitudes, and practices (KAP) related to SM among the general population in the central region of Saudi Arabia.

### 2.2 Study participants

The study targeted all residents aged 18 years and older living in urban and rural areas of the central region of Saudi Arabia, while excluding healthcare workers and anyone younger than 18 years old. Participants were recruited using a non-probability convenience sampling approach. The questionnaire was distributed electronically through online platforms and supplemented by in-person distribution to enhance reach across the target population. Participation was voluntary, and only eligible individuals who provided informed consent were included in the study.

### 2.3 Sample size calculation

The sample size was determined using the “Raosoft” online calculator. According to the General Authority for Statistics (14), the population of the central region of Saudi Arabia is approximately 10.6 million. To achieve a 95% confidence level with a 5% margin of error, a minimum sample size of 385 was required. Although 385 responses were initially collected, 384 valid records were retained for the final statistical analysis after data screening and cleaning.

### 2.4 Inclusion and exclusion criteria

By applying these criteria, we will ensure that the sample accurately reflects the research aims while reducing potential confounding factors that might influence the results.

Inclusion:

- Age: participants should be 18 years or older.
- Gender: both males and females.
- Location: residents of the central region of Saudi Arabia.
- Consent: participants willingly agree to participate and provide consent.
- Comprehension: participants can understand Arabic and/or English, fully comprehend the questions, and answer them accurately.

Exclusion:

- Healthcare workers: to avoid bias, as their medical background may influence their answers.
- Age: participants younger than 18 years.

### 2.5 Study questionnaire development

The study sample size was calculated following the study protocol (15), initially, the survey was distributed to a pilot sample of ten faculty members to countercheck the validity of the questionnaire and identify any technical concerns. We attempted to design a survey that was as succinct as possible. In addition, we explained that the information provided would be utilized only for research purposes. The study’s sampling frame comprised adults living in the central region of Saudi Arabia. Participants were recruited through a stratified sample method to guarantee representation from various demographic groupings within the population. Data were collected using a self-administered electronic questionnaire via Google Forms, distributed randomly in Saudi Arabia. Participants were primarily recruited through institutional mailing lists (36.4%), social media platforms such as Twitter and WhatsApp (39.1%), and direct peer-to-peer snowball sharing (24.5%). Although online surveys may over represent digitally literate and health-conscious individuals, the use of diverse recruitment strategies helped mitigate this bias by reaching participants. Google Form settings were used to stop duplicate responses. A validated survey was used to assess participants’ knowledge and behaviors regarding SM. A structured questionnaire was designed and disseminated via Google Forms, developed in both Arabic and English. The questionnaire consisted of four sections: demographic information, knowledge about SM, attitudes toward its use, and self-reported practices. It was distributed both electronically via online platforms and in person to ensure wide coverage. The distribution started on 06 June 2025 and ended on 11 December 2025. The questionnaire was reviewed by a panel of experts and non-experts for content validity. The knowledge domain comprised six items, the attitude domain comprised four items, and the practice domain comprised two items. Each item was rated on a scale of 1 to 3, with higher scores meaning higher levels in the domain. Domain scores were calculated as the mean of the corresponding item scores, resulting in a possible score range from (1.00) to (3.00) for knowledge, attitude, and practice. For interpretive purposes, mean scores were categorized into three levels using equal-interval classification: low (1.00)–(1.66), moderate (1.67)–(2.33), and high (2.34)–(3.00).

### 2.6 Statistical analysis

All statistical analyses were conducted using IBM SPSS Statistics version 28. Descriptive statistics, including frequencies, percentages, medians, and interquartile ranges (IQR), were used to summarize participants’ demographic characteristics and their responses across the knowledge, attitude, and practice (KAP) domains related to self-medication. Knowledge, Attitude, and Practice scores were computed by averaging the items within each domain. All items were measured on a three-point scale (1–3), yielding composite scores ranging from 1.00 to 3.00, with higher values indicating higher levels on the respective domain. Normality of the KAP scores was assessed across demographic groups using both the Kolmogorov and Shapiro–Wilk tests. All three domain scores showed significant deviations from normality (*p* < 0.05), supporting the use of nonparametric statistical methods. For demographic variables with two groups (gender and educational level), differences in Knowledge, Attitude, and Practice scores were evaluated using the Mann–Whitney U test. For variables with more than two categories (age groups and income levels), the Kruskal–Wallis H test was used. For all comparisons, mean ranks, median values, interquartile ranges, U, Z, H statistics, *p*-values, and effect sizes were reported, using r for Mann–Whitney U tests and epsilon-squared (ε^2^) for Kruskal–Wallis tests. Statistical significance was set at *p* < 0.05. All analyses were performed using complete cases with listwise deletion for missing data.

### 2.7 Ethical approval

The Institutional Review Board (IRB) approval and authorization were obtained from Imam Muhammad bin Saud Islamic University’s committee with project number 832/2025, which was validated on 25/06/2025. The study, as approved by the IRB, carried the title: “Knowledge, Attitude, and Practice of SM Among Adults in the Central Region of Saudi Arabia: A Cross-sectional study”.

## 3 Results

A total of 384 participants completed the questionnaire. Table 1 presents the demographic characteristics of the respondents. Females represented a slightly higher proportion of the sample (215; 56.0%), whereas males accounted for (169; 44.0%). The majority of participants were young adults aged 18–28 years (221; 57.6%). This cohort was followed by participants aged 40–50 years (65; 16.9%) and those aged 29–39 years (53; 13.8%). Participants aged above 50 years represented the smallest group (45; 11.7%). Most respondents were university graduates (273; 71.1%), while participants with a high school education or below constituted (111; 28.9%). Nearly half of the sample reported earning 3,000 Riyals or less (190; 49.5%). Those earning 3,001–7,000 Riyals constituted (49; 12.8%), while (35; 9.1%) earned 7,001–10,000 Riyals. Participants with incomes exceeding 10,000 Riyals accounted for (110; 28.6%). The majority of participants did not have any chronic conditions (323; 84.1%), whereas (61; 15.9%) indicated living with a chronic disease. Finally, the knowledge of SM, more than half of the respondents answered No (209; 54.4%), while (175; 45.6%) had heard of it before.

**Table 1:** Demographic characteristics of study participants (*n* = 384).

Variables	Frequency (*N*)	Percent (%)
Gender		
Male	169	44.0%
Female	215	56.0%
Age groups (years)		
18–28	221	57.6%
29–39	53	13.8%
40–50	65	16.9%
>50	45	11.7%
Education level		
High school or less	111	28.9%
University graduate	273	71.1%
Monthly income (SAR)		
3,000 or less	190	49.5%
3,001–7,000	49	12.8%
7,001–10,000	35	9.1%
More than 10,000	110	28.6%
Chronic diseases		
No	323	84.1%
Yes	61	15.9%
Heard of self-medication		
No	209	54.4%
Yes	175	45.6%

**Figure 1:**
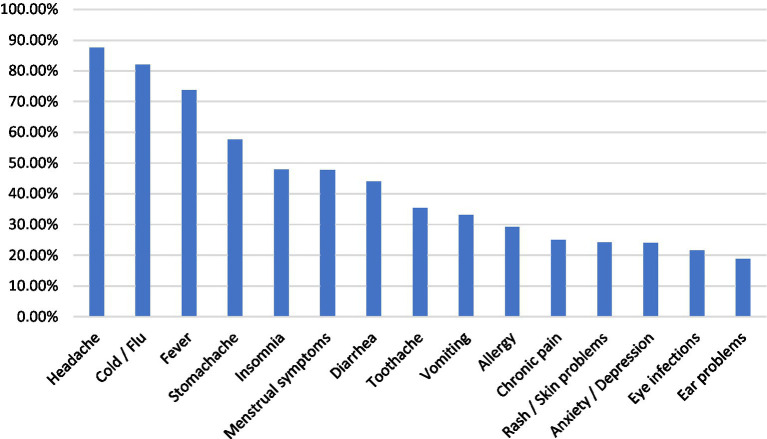
Indications for practicing self-medication among participants (multiple-response item; percentages are based on the total sample, *n* = 384). Multiple responses were allowed; therefore, percentages do not sum to 100%. Percentages were calculated using the total study sample (*n* = 384) as the denominator.

Figure 1 illustrates the symptoms and medical conditions that prompted participants to practice SM. The results show that headache was the most frequently reported reason for engaging in SM (336; 87.5%), followed by common cold and influenza (315; 82.0%) and fever (283; 73.7%). These findings indicate that participants most often resort to self-treatment for common, self-limiting ailments that typically do not require urgent medical intervention. Moderately prevalent indications included stomach-ache (221; 57.6%), insomnia (184; 47.9%), and menstrual symptoms (183; 47.7%).

**Figure 2:**
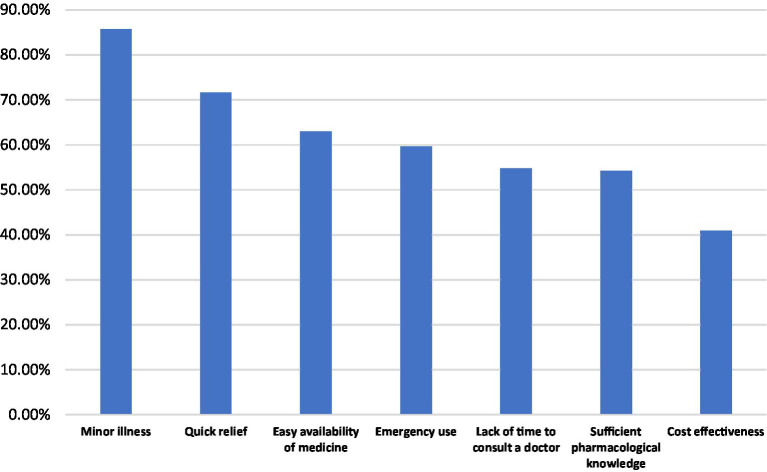
Reasons for practicing self-medication among participants (multiple-response item; percentages are based on the total sample, *n* = 384). Multiple responses were allowed; therefore, percentages do not sum to 100%. Percentages were calculated using the total study sample (*n* = 384) as the denominator.

Figure 2 presents the reasons that prompted participants to engage in SM. The results reveal that the most frequent was minor illness (329; 85.7%), indicating that individuals often view SM as an acceptable approach for managing simple, self-limiting health problems.

The second most common reason was the desire for quick relief (275; 71.6%), highlighting an inclination toward immediate symptom management without delays associated with formal healthcare visits. Additionally, easy availability of medicines (242; 63.0%) and the need for emergency use (229; 59.6%) were notable drivers, suggesting that accessibility and urgency play substantial roles in shaping self-treatment behaviors.

More than half of the participants reported lack of time to consult a doctor (210; 54.7%) and having sufficient pharmacological knowledge (208; 54.2%) as key motivating factors. These findings may reflect perceived convenience and confidence in personal health literacy. On the other hand, cost effectiveness (157; 40.9%) was the least reported reason, though still substantial, pointing to economic considerations as a secondary but relevant motivator.

**Figure 3:**
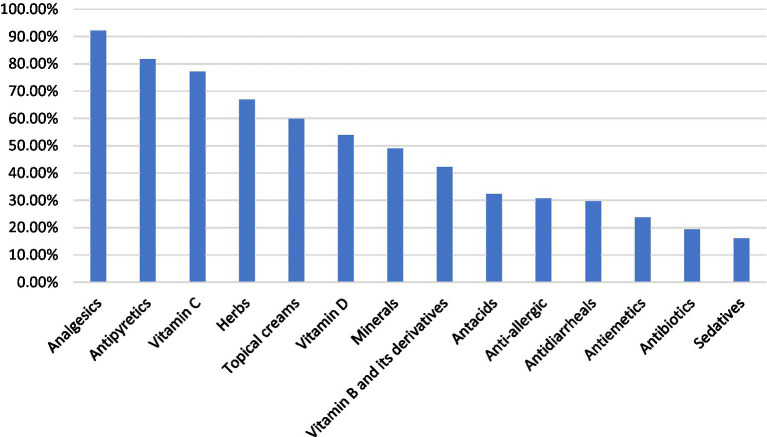
Types of self-prescribed medicines used by participants (multiple-response item; percentages are based on the total sample, *n* = 384). Multiple responses were allowed; therefore, percentages do not sum to 100%. Percentages were calculated using the total study sample (*n* = 384) as the denominator.

Figure 3 presents the distribution of medications commonly used by participants for SM. The most frequently utilized types were analgesics (354; 92.2%) and antipyretics (314; 81.8%), highlighting a strong reliance on over-the-counter treatments for pain and fever management. The use of herbal remedies was also prominent (257; 66.9%), reflecting cultural tendencies toward natural or traditional forms of treatment.

Nutritional supplements were widely reported, with Vitamin C being the most commonly used (296; 77.1%), followed by Vitamin D (207; 53.9%) and minerals such as iron, calcium, and magnesium (188; 49.0%). Use of Vitamin B and its derivatives was also substantial (162; 42.2%), suggesting a broad interest in supplementation as part of self-care practices.

Moderate levels of use were noted for topical creams (230; 59.9%) and antacids (124; 32.3%). Meanwhile, medications such as anti-allergic agents, antidiarrheals, and antiemetics were used by approximately one-quarter to one-third of the participants, indicating targeted use for specific symptoms.

On the lower end, potentially high-risk medications such as antibiotics (74; 19.3%) and sedatives (62; 16.1%) were reported less frequently than other medication classes. However, this finding should be interpreted cautiously, as even relatively modest non-prescription antibiotic use may contribute to antimicrobial resistance and remains a relevant public health concern.

Table 2 summarizes the factors that motivated participants to practice SM. The most influential factor was personal experience (324; 84.4%), indicating that individuals rely heavily on their familiarity with previous illnesses and treatments when deciding to SM. This suggests that experiential learning plays a central role in shaping self-care behaviors. The influence of family members’ opinions was also substantial (272; 70.8%), reflecting the strong impact of family dynamics and social support within the cultural context. Likewise, more than half of the participants reported being influenced by a previous prescription from a doctor (251; 65.4%), highlighting a tendency to reuse earlier medical advice or medications without seeking new professional consultation. Nearly half of the respondents cited friends’ opinions (186; 48.4%) as a contributing factor, suggesting that peer networks play a moderate yet notable role in shaping decisions regarding SM. In contrast, advertising had the least influence (53; 13.8%), indicating that commercial promotional materials are not a primary driver of SM behavior among this population.

**Table 2:** Factors influencing participants’ decision to practice self-medication (*n* = 384).

Influencing factor	Frequency (*N*)	Percent (%)
Own experience	324	84.4%
Family member’s opinion	272	70.8%
Previous prescription from a doctor	251	65.4%
Friend’s opinion	186	48.4%
Advertisement	53	13.8%

Table 3 presents the nonparametric comparison of Knowledge scores across demographic groups. A Mann–Whitney U test revealed a statistically significant difference between males and females (U = 16,069.5, Z = −1.969, *p* = 0.049), with females showing slightly higher knowledge scores (Median = 2.50, IQR = 2.17–2.67) than males (Median = 2.33, IQR = 2.17–2.50); however, the effect size was small (r = 0.101). No statistically significant difference was observed across educational levels (U = 14,402.0, Z = −0.770, *p* = 0.441), indicating similar knowledge scores between participants with high school education or below and university graduates; the effect size was negligible (r = 0.039). A statistically significant difference was observed across age groups (H = 10.848, *p* = 0.013). Participants aged 40–50 had the highest median knowledge score (Median = 2.50, IQR = 2.33–2.67), whereas those aged 29–39 had the lowest mean rank (Median = 2.33, IQR = 2.00–2.50); however, the effect size was small (ε^2^ = 0.021). No statistically significant differences were observed across income categories (H = 3.587, *p* = 0.310), indicating that knowledge scores did not differ significantly by monthly income; the effect size was negligible (ε^2^ = 0.002).

**Table 3:** Nonparametric comparison of knowledge scores across demographic groups (*n* = 384).

Variable	Group	*N*	Mean rank	Median *(IQR)*	Test Statistic U *(Z)* or H	*p*-value	Effect size
Gender	Male	169	180.09	2.33 (2.17–2.50)	U = 16,069.5 (Z = − 1.969)*	0.049	r = 0.101
	Female	215	202.26	2.50 (2.17–2.67)			
Education level	High school or below	111	185.75	2.33 (2.17–2.50)	U = 14,402.0 (Z = − 0.770)*	0.441	r = 0.039
	University graduate	273	195.25	2.33 (2.17–2.67)			
Age	18–28	221	190.34	2.33 (2.17–2.67)	H = 10.848**	0.013	ε^2^ = 0.021
	29–39	53	167.08	2.33 (2.00–2.50)			
	40–50	65	229.26	2.50 (2.33–2.67)			
	>50	45	179.97	2.33 (2.17–2.50)			
Monthly income (SAR)	3,000 or less	190	188.78	2.33 (2.17–2.67)	H = 3.587**	0.310	ε^2^ = 0.002
	3,001–7,000	49	212.41	2.50 (2.17–2.67)			
	7,001–10,000	35	169.56	2.33 (2.00–2.67)			
	>10,000	110	197.35	2.33 (2.17–2.67)			

*Mann–Whitney U test.

**Kruskal–Wallis H test.

Dependent variable: Knowledge.

Values represent the median (IQR), mean ranks, and test statistics for the nonparametric analyses. The Mann–Whitney U test was used for comparisons between two groups, whereas the Kruskal–Wallis H test was used for comparisons across three or more groups. *p* < 0.05 indicates statistical significance.

The nonparametric comparison presented in Table 4 showed no statistically significant differences in attitude toward SM across any demographic variables. A Mann–Whitney U test indicated no statistically significant difference between males and females (U = 17,775.0, Z = −0.377, *p* = 0.706), with both groups showing similar median attitude scores; the effect size was negligible (r = 0.019). Likewise, no statistically significant difference was observed across educational levels (U = 15,129.5, Z = −0.023, *p* = 0.982), indicating similar attitude scores between participants with high school education or below and university graduates; the effect size was negligible (r = 0.001). The Kruskal–Wallis H test also showed no statistically significant differences across age groups (H = 2.236, *p* = 0.525) or monthly income categories (H = 1.470, *p* = 0.689), with negligible effect sizes in both comparisons (ε^2^ = 0.002 and ε^2^ = 0.004, respectively). Overall, median attitude scores were similar across all demographic subgroups.

**Table 4:** Nonparametric comparison of differences in attitude across demographic groups (*n* = 384).

Variable	Group	N	Mean Rank	Median *(IQR)*	Test Statistic U *(Z)* or H	*p*-value
Gender	Male	169	194.82	2.25 (2.25–2.50)	U = 17,775.0 (Z = − 0.377)*	0.706
	Female	215	190.67	2.25 (2.00–2.50)		
Education Level	High school or below	111	192.30	2.25 (2.00–2.50)	U = 15,129.5 (Z = −0.023)*	0.982
	University graduate	273	192.58	2.25 (2.00–2.50)		
Age	18–28	221	197.17	2.25 (2.25–2.50)	H = 2.236**	0.525
	29–39	53	191.07	2.25 (2.00–2.50)		
	40–50	65	192.62	2.25 (2.00–2.50)		
	>50	45	171.09	2.25 (2.13–2.25)		
Monthly income (SAR)	3,000 or less	190	195.86	2.25 (2.00–2.50)	H = 1.470**	0.689
	3,001–7,000	49	184.31	2.25 (2.00–2.50)		
	7,001–10,000	35	206.30	2.25 (2.25–2.50)		
	>10,000	110	185.96	2.25 (2.00–2.50)		

*Mann–Whitney U test.

**Kruskal–Wallis H test.

Dependent variable: Attitude.

Values represent the median (IQR), mean ranks, and test statistics for the nonparametric analyses.

The Mann–Whitney U test was used for comparisons between two groups, whereas the Kruskal–Wallis H test was used for comparisons across three or more groups.

*p* < 0.05 indicates statistical significance.

Table 5 presents the results of the nonparametric comparison of SM practice across demographic groups. A statistically significant difference was found between males and females (U = 16,150.5, Z = −2.105, *p* = 0.035), with females showing slightly higher practice scores (Median = 2.00, IQR = 2.00–2.00) than males (Median = 2.00, IQR = 1.00–2.00); however, the effect size was small (r = 0.107). No statistically significant difference was observed across educational levels (U = 13,804.5, Z = −1.539, *p* = 0.124), indicating similar practice scores between participants with a high school education or below and university graduates; the effect size was negligible (r = 0.079). Likewise, the Kruskal–Wallis H test showed no statistically significant differences in practice across age groups (H = 1.669, *p* = 0.644) or monthly income categories (H = 0.444, *p* = 0.931), with negligible effect sizes in both comparisons (ε^2^ = 0.004 and ε^2^ = 0.007, respectively). Overall, no statistically significant differences in practice scores were observed across most demographic categories.

**Table 5:** Nonparametric comparison of differences in practice across demographic groups (*n* = 384).

Variable	Group	*N*	Mean rank	Median *(IQR)*	Test statistic U *(Z)* or H	*p*-value	Effect size
Gender	Male	169	180.57	2.00 (1.00–2.00)	U = 16,150.5 (Z = − 2.105)*	0.035	r = 0.107
	Female	215	201.88	2.00 (2.00–2.00)			
Education level	High school or below	111	180.36	2.00 (1.00–2.00)	U = 13,804.5 (Z = −1.539)*	0.124	r = 0.079
	University graduate	273	197.43	2.00 (2.00–2.00)			
Age	18–28	221	193.33	2.00 (2.00–2.00)	H = 1.669**	0.644	ε^2^ = 0.004
	29–39	53	199.93	2.00 (1.75–2.00)			
	40–50	65	195.22	2.00 (1.25–2.00)			
	>50	45	175.74	2.00 (1.00–2.00)			
Monthly income (SAR)	3,000 or less	190	193.86	2.00 (2.00–2.00)	H = 0.444**	0.931	ε^2^ = 0.007
	3,001–7,000	49	184.26	2.00 (1.00–2.00)			
	7,001–10,000	35	190.27	2.00 (1.50–2.00)			
	>10,000	110	194.53	2.00 (1.50–2.00)			

*Mann–Whitney U test.

**Kruskal–Wallis H test.

Dependent variable: Practice.

Values represent the median (IQR), mean ranks, and test statistics for the nonparametric analyses. The Mann–Whitney U test was used for comparisons between two groups, whereas the Kruskal–Wallis H test was used for comparisons across three or more groups. *p* < 0.05 indicates statistical significance.

## 4 Discussion

Since self-medication (SM) is a common practice in Saudi Arabia, concerns have been raised regarding the increasing risks associated with medication misuse, incorrect self-diagnosis, and delays in seeking appropriate medical care. This cross-sectional study was conducted to provide a comprehensive overview of SM behaviors and the associated knowledge, attitudes, and practices within the present sample. The novelty of this study lies in its inclusion of a broader range of medication classes, such as antibiotics, anti-allergic medications, and sedatives. In addition, the study examined SM practices involving dietary supplements, including vitamins and herbal products. Overall, the study sought to evaluate knowledge, attitudes, and practices related to SM in the study sample.

In Saudi Arabia, medications are classified by the Saudi Food and Drug Authority (SFDA) into over-the-Counter (OTC) medicines and prescription-only medicines (POM). The classification depends on factors such as safety margin, risk of misuse, suitability for self-diagnosis, and simplicity of use instructions. OTC medicines are non-prescription drugs that are safe for self-use and are not merely complementary products like vitamins or minerals. Some POMs can be reclassified as OTC if they meet criteria including a wide safety margin, safe self-diagnosis, simple instructions, and sufficient market experience among consumers.

OTC medicines may be advertised publicly across all media platforms with SFDA approval, while POMs are strictly prohibited from public advertising. All medications in Saudi Arabia are tracked using a GS1 Data Matrix barcode system, which allows the SFDA to monitor for misuse or abuse and to establish or update regulations as needed. This system has been applied to drugs such as antibiotics, GLP-1 analogs, and isotretinoin (Accutane) to ensure safe distribution and usage (16).

The demographic profile of the participants showed a predominance of females and young adults, with most respondents having attained a university level of education. This distribution should be interpreted cautiously, as it reflects the composition of the study sample rather than the broader adult population of the central region of Saudi Arabia. The relatively low proportion of participants reporting chronic illnesses may indicate that SM in this sample was more commonly reported in relation to short-term and self-limiting conditions rather than long-term disease management.

Knowledge about SM was generally moderate, with more than half of the respondents indicating limited prior awareness of the concept. This finding highlights a gap between the widespread practice of SM and formal understanding of its definition and potential risks. Gender differences emerged in knowledge levels, with females demonstrating slightly higher awareness. This may reflect greater health-seeking behavior, increased exposure to health information, or more frequent engagement in self-care activities among females. Age-related differences were also observed, with middle-aged participants exhibiting higher knowledge levels than younger adults, possibly due to accumulated life experience and repeated interactions with healthcare systems. In contrast, educational attainment and income level did not appear to significantly influence knowledge, suggesting that awareness of SM is shaped more by experiential factors than by formal education or socioeconomic status.

Attitudes toward SM did not differ significantly across the demographic groups. Examined. No statistically significant differences were observed by gender, age, education, or income indicating similar attitude scores across these subgroups. These findings should be interpreted cautiously and within the context of the study sample, particularly given the demographic composition of the participants and the absence of multivariable analysis.

Practice patterns revealed that SM was most commonly undertaken for symptoms such as headache, respiratory infections, and fever. These conditions are generally perceived as minor and self-limiting, which explains the high reliance on self-treatment rather than professional medical care. Gastrointestinal discomfort, sleep disturbances, and menstrual symptoms were also frequent triggers, further illustrating the tendency to self-manage recurring or non-urgent health issues. The preference for SM in these contexts reflects a pragmatic approach to symptom relief and highlights the importance of accessibility and immediacy in health decision-making.

Pharmacist-led interventions could have a significant impact on SM practices. The role of a pharmacist in SM is important, as was explained by the International Pharmaceutical Federation (FIP). Pharmacists can educate patients about drug indications, dosage and duration, and potential side effects and interactions with other drugs. This would aim to improve patient knowledge and awareness, and would most likely result in a reduction of improper SM and the promotion of safe SM. Repeated interactions with pharmacists can instill habits of consulting with medical professionals before taking medications, instead of relying on anecdotal advice or advice from the internet.

To situate these findings within a broader regional context without shifting the study’s focus, evidence from Arab settings with comparable access to community pharmacies suggests a similar “minor illness–convenience” pattern of SM. During the COVID-19 period, a multinational survey across Arab countries reported widespread SM and common medicine classes, reflecting routine management of self-limiting symptoms (17). In Egypt, community-based data from Alexandria highlight headache/body aches, flu/common cold/cough, and fever among frequently reported indications for SM (18). The nationwide Egyptian survey also documented substantial (SM) and reported antibiotics among commonly used medication classes [101]. These comparisons are provided only to contextualize the Saudi findings, while acknowledging that prevalence estimates may vary across studies due to differences in sampling, recall periods, and questionnaire formats (17–19).

A study by Almalki et al. (15) on SM use among the western region of Saudi Arabia found that out of 647 survey respondents, 67.7% practiced SM. While another study conducted in 2022 on SM practices and perceptions in Riyadh, by Mannasaheb et al. (20) which showed that 52.9% practice SM. Both Mannasaheb et al., and Almalki et al., agreed with the present study, that due to easy availability of medicines for minor illnesses such as headache, cold/flu, and fever. Very similar to Almalki et al. (15) study, in the present study females showed higher engagement on SM practice. Both studies agrees that the major usage of SM was on incidents includes headache, cold/flu (42.7%), and fever.

Analgesics, antipyretics, and herbal remedies were the most frequently used medications, indicating substantial reliance on over-the-counter and traditional products. The widespread use of vitamins and nutritional supplements may reflect a broader perception of SM as part of preventive health and general well-being, rather than solely as a response to illness. Although antibiotics and sedatives were reported less frequently than other medication classes, this finding should be interpreted cautiously, as even relatively modest non-prescribed use of such medications remains a relevant public health concern because of the potential risks of antimicrobial resistance and dependency.

From a public health perspective, the reported antibiotic SM rate in the current sample (19.3%) warrants cautious interpretation. Although this proportion appears lower than that reported in some neighboring (e.g., Egypt) (18, 19), even modest non-prescribed antibiotic use may contribute to antimicrobial resistance. These findings underscore the continued need for public awareness campaigns, stricter stewardship of antibiotic dispensing, and pharmacist-led education to promote the rational use of antimicrobials.

In 2018, the Saudi Ministry of Health implemented a nationwide antibiotic restriction policy, classifying all antibiotics as Prescription-Only Medicines (POM) and introducing penalties, including fines and pharmacy licensing actions, for dispensing without a valid prescription. This regulation aimed to reduce antibiotic misuse and combat antimicrobial resistance (AMR), as a common misconception in Saudi society was that antibiotics could be used for any infection, including viral infections and the flu, without consulting a healthcare provider. Such misuse had contributed to an increase in resistant bacteria, which are more difficult to treat (21, 22).

In addition to regulatory measures, the SFDA conducted multiple education and awareness campaigns to teach the public about proper antibiotic use and the potential complications of misuse. These initiatives highlighted the individual, national, and global dangers of AMR, emphasizing that responsible antibiotic use is critical to protecting public health worldwide (23).

The primary motivations for SM cantered on the perception of illness as minor and the desire for rapid symptom relief. Factors such as easy access to medications, urgency of symptoms, lack of time to seek medical care, and confidence in personal pharmacological knowledge further reinforced SM behaviors. Similar to present study, in 2024 a study conducted by Abdulrahman et al. stated that around 62% seek SM to “save time.” Economic considerations played a secondary role, indicating that convenience and perceived efficiency are more influential than cost alone. These findings emphasize how modern lifestyles and healthcare accessibility shape self-care practices. Nonetheless, Khalid et al. found a correlation between monthly income and SM practice (24). Higher monthly income was a major factor, around 91.2% rate, this can lead to the enhanced access to pharmaceuticals and the ability to easily buy them.

Social influences were also significant determinants of SM. Personal experience emerged as the strongest motivating factor, highlighting the role of trial-and-error learning in health behavior. Family members and previous medical prescriptions exerted considerable influence, suggesting that individuals often rely on informal advice and past professional guidance rather than seeking new consultations. In contrast, advertising had minimal impact, indicating that SM decisions are driven more by trust in familiar sources than by commercial messaging.

Abdulrahman et al. (24), study was based in Riyadh, Saudi Arabia, limited to only university students at public universities in Riyadh evaluating SM. This study also showed that the majority of the participants (81%) practice SM. These students varied between majors therefore the accessibility to students in the medical fields including pharmacists was high. This study indicated that participants who were affiliated with medical colleagues show higher SM practice (62.3%) compared to those that were not affiliated with medical colleagues (46.8%).

Gender differences were evident in SM practice, with females reporting slightly higher engagement. This aligns with existing literature suggesting that women are more proactive in managing health concerns and more likely to use medications for symptom relief. However, no statistically significant differences were observed across age, education, or income groups in practice scores, and these findings should be interpreted within the context of the study sample.

Alghanim (12) examined SM in 500 Riyadh primary healthcare patients. SM (35.4%) for minor illnesses like 72.40%, fever (55.20%), and common cold (65.50%) using OTC analgesics and antipyretics was lower in the previous two weeks, consistent with this study. Like this study, easy access to medications, lack of time to visit healthcare facilities (40%), and prior personal experience (50.10%) were the main reasons for SM. Males had a higher prevalence (70%) than females, likely due to study population, time frame, and social context (males obtaining medication for women). This study matches Saudi Arabia’s SM reasons and indications despite prevalence and demographic differences (12).

Comparing the findings with Albusalih et al. (13), who studied 300–400 pharmacy and medical students at a public university in Dammam, Saudi Arabia, on SM prevalence and patterns. SM for minor illnesses like headache, fever, pain, and dysmenorrhea (14.2%) was common among university students. Most medications used were analgesics and antipyretics (72.35%). SM was driven by minor illness (35.1%) and prior experience (14.2%) in both populations. This study was limited to university students, but the results reflect the general pattern of SM, emphasizing the need for educational interventions to promote rational drug use and raise awareness of the risks of unsupervised medication (13).

## 5 Limitations

This study has several limitations related to the assessment of knowledge, attitudes, and practices regarding SM among the general population in the central region of Saudi Arabia. First, the sampling approach relied largely on convenience sampling through online distribution, which may have introduced selection bias. Individuals who are more active on digital platforms or more interested in health-related topics may have been more likely to participate. Consequently, the sample may over-represent younger and more highly educated participants, which could influence the overall estimates of knowledge, attitudes, and practices related to SM. Second, the study relied on self-reported data collected through a self-administered questionnaire. This approach may have introduced information bias, as respondents could overestimate or underestimate their actual level of knowledge and their SM behaviors. Self-reported responses are also subject to recall bias, particularly when participants were asked to report the frequency, type of medications used, and duration of SM. In addition, some participants may have underreported certain behaviors, such as the use of prescription-only medications without medical consultation, due to social desirability bias. Third, knowledge regarding SM was assessed using predefined questionnaire items. Although these questions were designed to capture key aspects of medication knowledge, they may not fully reflect the depth of participants’ understanding, including practical decision-making, awareness of drug–drug interactions, or the ability to recognize potentially harmful medication use. Moreover, the knowledge, attitude, and practice (KAP) assessment did not rely on a previously validated scoring system, which may affect the precision and comparability of the measurements. Fourth, attitudes toward SM are inherently subjective and may vary depending on personal experiences, recent illness episodes, or interactions with healthcare providers. The responses provided by participants may therefore reflect socially acceptable views rather than their actual beliefs or behaviors. Furthermore, the cross-sectional design captures attitudes and practices at a single point in time and does not allow for the evaluation of changes over time. Another limitation is that the statistical analysis did not include multivariable adjustment for potential confounding variables that may influence SM behavior, such as socioeconomic status, access to healthcare services, or the presence of chronic illness. As a result, the observed associations between knowledge, attitudes, and practices should be interpreted with caution. Finally, the study was conducted in the central region of Saudi Arabia, and therefore the findings may not be fully generalizable to populations in other regions of the country that may differ in healthcare accessibility, cultural practices, and educational backgrounds. Despite these limitations, the study provides valuable baseline information about SM behaviors in the community and may help inform future research and targeted public health interventions.

## 6 Conclusion

In this sample of adults from the central region of Saudi Arabia, SM appeared to be a common health behavior, largely associated with perceptions of minor disease, convenience, prior personal experience, and social influences. No statistically significant differences were observed across several demographic comparisons in knowledge and attitude scores. These findings should be interpreted cautiously, particularly given the absence of multivariable analysis and the overrepresentation of younger adults and university graduates in the sample. Nevertheless, the findings highlight the need for targeted public health education to promote safer and more informed SM practices, particularly regarding appropriate medication use, potential risks, and the importance of professional consultation when needed.

## Data Availability

The original contributions presented in the study are included in the article/supplementary material, further inquiries can be directed to the corresponding author.

## References

[1] Hughes CM McElnay JC Fleming GF. Benefits and risks of self medication. Drug Saf. (2001) 24:1027–37. doi: 10.2165/00002018-200124140-00002 11735659

[2] Ruiz ME. Risks of self-medication practices. Curr Drug Saf. (2010) 5:315–23. doi: 10.2174/157488610792245966, 20615179

[3] Bennadi D. Self-medication: a current challenge. J Basic Clinical Pharm. (2013) 5:19–23. doi: 10.4103/0976-0105.128253, 24808684 PMC4012703

[4] Loyola Filho AI Uchoa E Firmo JOA Lima-Costa MF. A population-based study on use of medications by elderly Brazilians: the Bambuí health and aging study (BHAS). Cad Saude Publica. (2005) 21:545–53. doi: 10.1590/S0102-311X2005000200021 15905917

[5] Ritz L.S., Access to Medicines Publications in Developing Countries: A Bibliometric Study and its Implications for the Access to Medicines Research Network. (2025).

[6] Sharma R Verma U Sharma CL Kapoor B. Self-medication among urban population of Jammu city. Indian J Pharmacol. (2005) 37:40–3. doi: 10.4103/0253-7613.13856

[7] Lukovic JA Miletic V Pekmezovic T Trajkovic G Ratkovic N Aleksic D . Self-medication practices and risk factors for self-medication among medical students in Belgrade, Serbia. PLoS One. (2014) 9:e114644. doi: 10.1371/journal.pone.0114644, 25503967 PMC4263675

[8] Aljadhey H Assiri GA Mahmoud MA al-Aqeel S Murray M. Self-medication in Central Saudi Arabia: community pharmacy consumers’ perspectives. Saudi Med J. (2015) 36:328–34. doi: 10.15537/smj.2015.3.10523, 25737176 PMC4381018

[9] Ventola CL. The antibiotic resistance crisis: part 1: causes and threats. Pharmacy Therapeutics. (2015) 40:277–83. 25859123 PMC4378521

[10] Rehman M Ahmed S Ahmed U Tamanna K Sabir MS Niaz Z. An overview of self-medication: a major cause of antibiotic resistance and a threat to global public health. JPMA. The. J Pak Med Assoc. (2021) 71:943–9. doi: 10.47391/jpma.1331 34057954

[11] (WHO), W.H.O., Antimicrobial Resistance. (2020).

[12] Alghanim S. Self-medication practice among patients in a public health care system. East Mediterr Health J. (2011) 17:409–16. doi: 10.26719/2011.17.5.409 21796954

[13] Albusalih FA Naqvi AA Ahmad R Ahmad N. Prevalence of self-medication among students of pharmacy and medicine colleges of a public sector university in Dammam City, Saudi Arabia. Pharmacy. (2017) 5:51. doi: 10.3390/pharmacy5030051 28970463 PMC5622363

[14] Statistics., G.A.f., General Authority for Statistics. Retrieved. (2025).

[15] Almalki ME Almuqati FS Alotaibi MO Makki SY Alqasem MA Alsharif FF . A cross-sectional study of the knowledge, attitude, and practice of self-medication among the general population in the western region of Saudi Arabia. Cureus. (2022) 14:e29944. doi: 10.7759/cureus.29944 PMC9635938 36381834

[16] Arabia, T.P.L.H.S., PharmaBoardroom. Prepared in Association with STA. (2021).

[17] Abdelwahed AE Abd-elkader MM Mahfouz A Abdelmawla MO Kabeel M Elkot AG . Prevalence and influencing factors of self-medication during the COVID-19 pandemic in the Arab region: a multinational cross-sectional study. BMC Public Health. (2023) 23:180. doi: 10.1186/s12889-023-15025-y, 36707840 PMC9880368

[18] El-Nimr N. Self-medication with drugs and complementary and alternative medicines in Alexandria, Egypt: prevalence, patterns and determinants. East Mediterr Health J. (2015) 21:256–65. doi: 10.26719/2015.21.4.256, 26077520

[19] Ali HT Barakat M Abdelhalim AR al-Kurd IN Muhammad MKE Sharkawy MM . Unravelling the dilemma of self-medication in Egypt: a cross-sectional survey on knowledge, attitude, and practice of the general Egyptian population. BMC Public Health. (2024) 24:652. doi: 10.1186/s12889-024-17913-3, 38429721 PMC10905903

[20] Mannasaheb BA Alajlan SA Alshahrani JA Othman N Alolayan SO Alamrah MS . Prevalence, predictors and point of view toward self-medication among residents of Riyadh, Saudi Arabia: a cross-sectional study. Front Public Health. (2022) 10:862301. doi: 10.3389/fpubh.2022.862301, 35400077 PMC8989923

[21] Alajel SM Alzahrani KO Almohisen AA Alrasheed MM Almomen SM. Antimicrobial sales comparison before and after the implementation of nationwide restriction policy in Saudi Arabia. Antibiotics. (2023) 13:15. doi: 10.3390/antibiotics13010015, 38275325 PMC10812388

[22] Bin Abdulhak AA al Tannir MA Almansor MA Almohaya MS Onazi AS Marei MA . Non prescribed sale of antibiotics in Riyadh, Saudi Arabia: a cross sectional study. BMC Public Health. (2011) 11:538. doi: 10.1186/1471-2458-11-538, 21736711 PMC3146870

[23] Authority, S.F.a.D., “SFDA” Launches Awareness Campaign for the Optimal use of Antibiotics. (2014)

[24] Abdulrahman KB Alharbi AK Alhaddad AM Alshaya AS Aldayel AS Aljumaiah MA. Self-medication practices among university students at a public university in Riyadh, Saudi Arabia. J Family Med Prim Care. (2024) 13:3773–81. doi: 10.4103/jfmpc.jfmpc_308_24, 39464905 PMC11504791

